# Congenital hyporhinia with associated malformations: Case report of a rare congenital anomaly

**DOI:** 10.1002/ccr3.7099

**Published:** 2023-03-15

**Authors:** Kenneth Mlay, Desderius Chussi, Philbert Mtenga, Peter Shija, Angela Msele

**Affiliations:** ^1^ Kilimanjaro Christian Medical Centre (KCMC) Moshi Tanzania; ^2^ Department of Otorhinolaryngology, Head and Neck Surgery Kilimanjaro Christian Medical University College (KCMUCo) Moshi Tanzania

**Keywords:** cebocephaly, congenital hyporhinia, holoprosencephaly, partial arhinia

## Abstract

Congenital hyporhinia, also known as partial arhinia, is a very rare congenital abnormality of nasal embryogenesis with unknown etiology. It is commonly associated with other craniofacial anomalies which are thought to be caused by an absent or rudimentary nose. A 3‐h‐old neonate presented to our facility with hypoplastic nasal pyramid, hypertelorism, microcephaly, and micrognathia, a case of congenital hyporhinia with associated anomalies is presented and the embryology and literature review are discussed.

## 1 INTRODUCTION

Congenital partial arhinia or hyporhinia is very uncommon congenital nasal anomaly. Less than 40 cases of congenital arhinia have been reported in the available literatures, most of them defined as complete arhinia and only four cases were congenital hyporhinia.^1^ The cause is not clearly known and most cases are sporadic, but there are few familial cases which have been reported.^2^ This pathology is usually found associated with other malformations affecting central nervous system, craniofacial, ear defects, and palatal clefts. Airway, feeding, and phonetic problems are usually accompanying this pathology in children.^3^ High mortality rate is commonly associated with this congenital malformation. We report an extremely rare case of a congenital absence of the heminose (partial arhinia) in a 3‐h‐old girl with other associated anomalies. To the best of our knowledge, this is among the few cases of heminose agenesis with associated malformations to be reported in the carefully reviewed literatures.

## 2 CASE HISTORY

Attention of otorhinolaryngologist was drawn to review a 2‐h old full‐term female neonate born from 17 years prime gravid, presented with nasal malformation, respiratory distress, and bluish coloration, delivered vaginally with APGAR score 5 and 6 at 1st and 5th minute at a GA 36 weeks. Pregnancy was supervised with reported 3 antenatal visits, screened for malaria, syphilis, and HIV which was negative. She received all important supplements, and no complications or medical conditions were brought into attention throughout pregnancy. Father 22 years and were not consanguineously married, no familial history of congenital malformations. Mother had no history of ingestion of any traditional medicines or exposure to radiation during her pregnancy; she does not smoke or drink alcohol and has never abused drugs.

**FIGURE 1:**
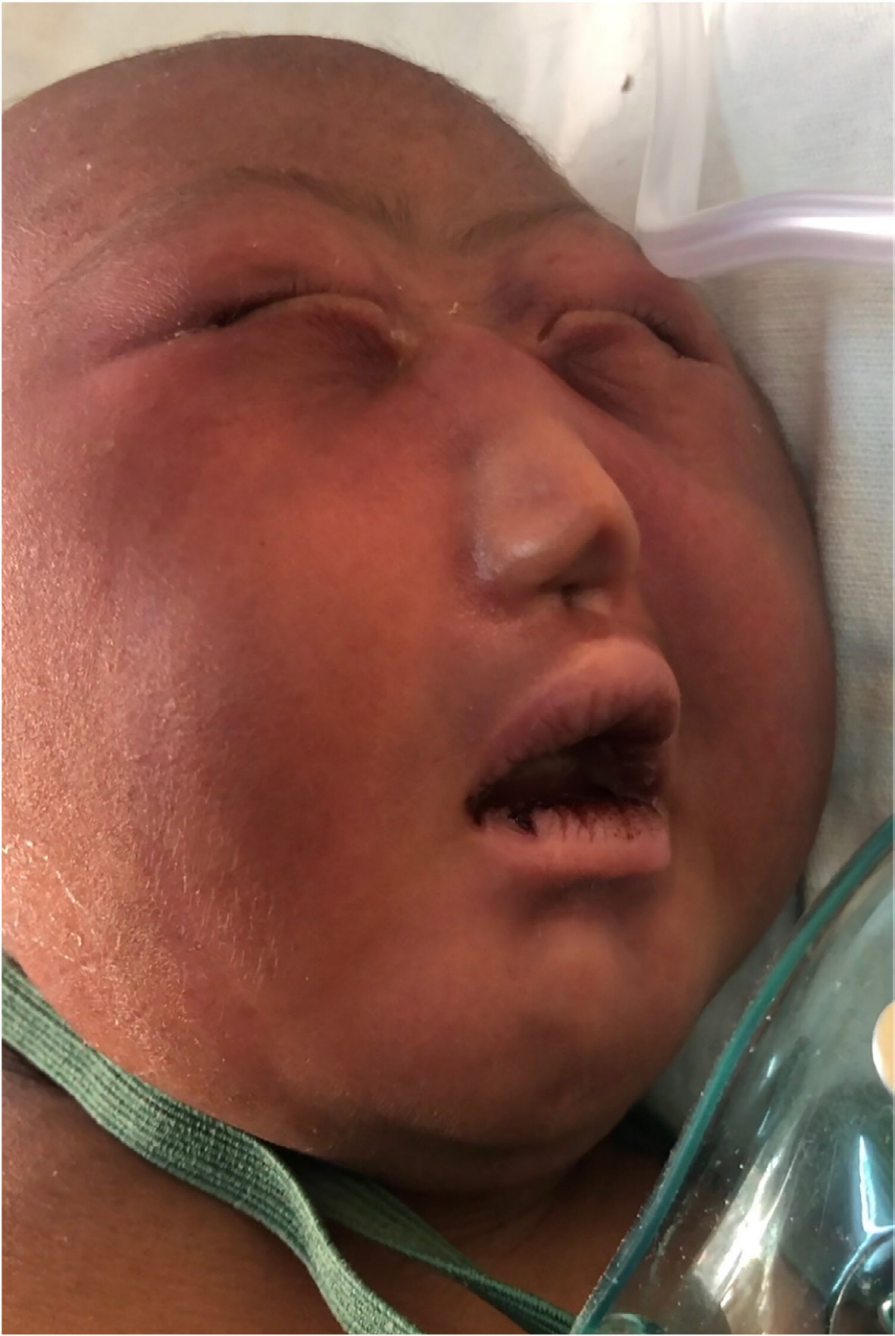
Picture of neonate showing hyporhinia with microcephaly and hypotelorism.

Examination revealed birth weight of 3.2 kg with microcephaly and had single nostril with remnants of alar cartilage barely palpable and a centrally placed single stenotic anterior nasal nare with pinpoint perforation. Columella, nasal septum, and the philtrium were absent. Also, she had microcephaly, high arched palate, and hypotelorism (Figure 1).

**FIGURE 2:**
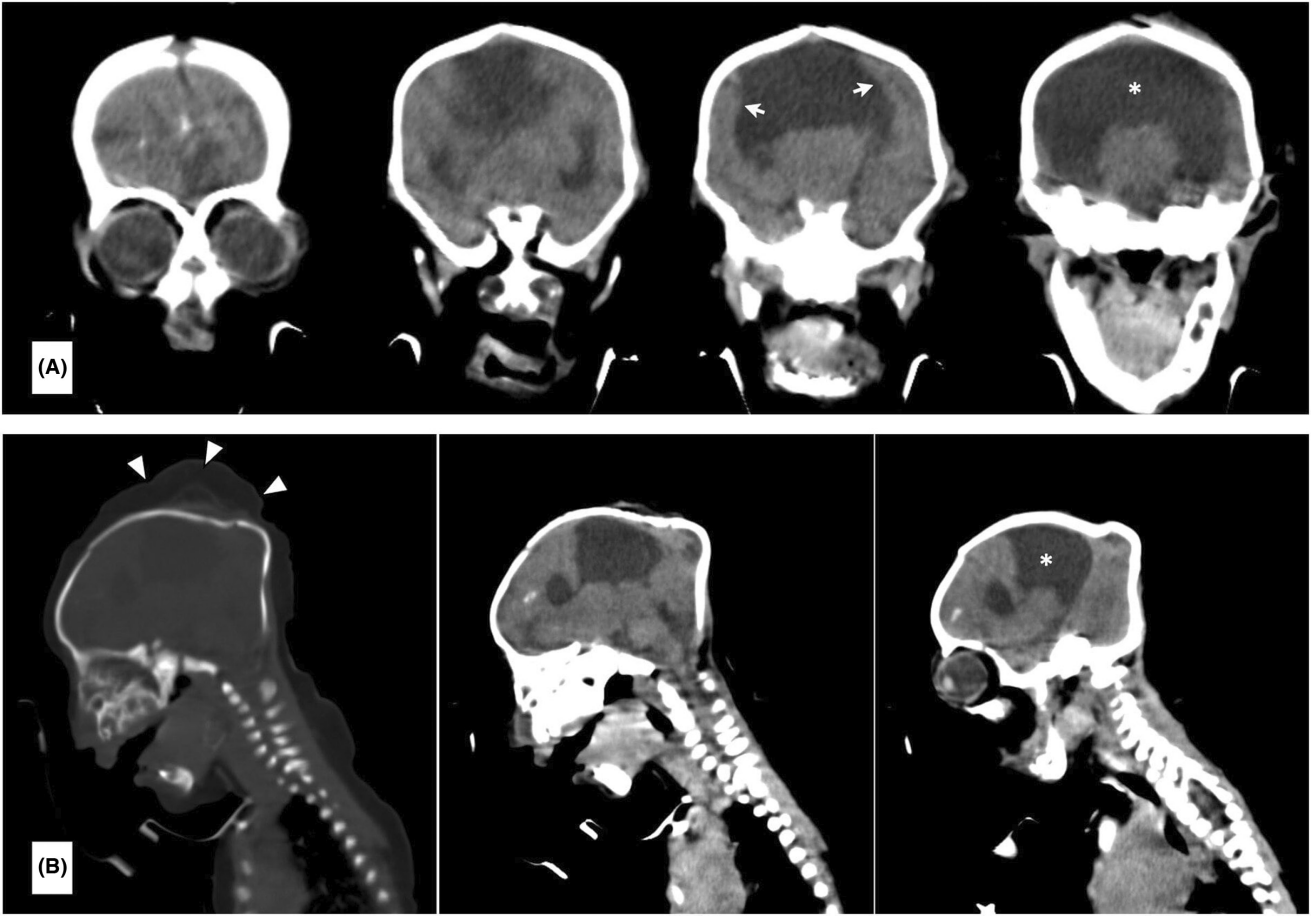
Non‐contrast CT scan of the brain in coronal (A) and sagittal (B) views showing a monoventricle (asterisk), fused cortex (white arrows), and cephalohematoma of the vertex (white arrowheads).

Initial stabilization was provided by maintenance of oral airway, and oxygenation. Trial to insert nasal gastric tube through the single nostril done which revealed anterior nare leading to 4‐mm‐deep single nasal cavity with atretic posterior choanae. There was ongoing discussion about stent insertion and tracheostomy, since some family members were hesitating for surgical intervention. Echocardiography was done and did not reveal any abnormality, chromosomal analysis to delineate extent and cause of malformation was not done. Non‐contrast computerized tomography scan of the brain (Figure 2) revealed head and nose to be small with a cephalohematoma in the vertex and an intranasal soft tissue density lesion blocking the entrance measuring approximately 10 × 8 mm. Absence of the corpus callosum and septum pellucidum with a resulting monoventricle formed from the lateral ventricles. And meanwhile, the baby continued to be nursed in neonatal ward she succumbed to death 6 days post‐admission due to severe respiratory failure.

## 3 DISCUSSION

Congenital hyporhinia is a rare defect of embryogenesis which is associated with other craniofacial abnormalities such as holoprosencephaly and midline defects.^4^

The patient had holoprosencephaly with cebocephaly, microcephaly, and high arched palate. Orbital anomalies are frequently associated with this condition,^5^ and our index case had hypotelorism.

Clinical presentation depends on the severity of nasal hypoplasia, and patient with atretic posterior choanae present with severe respiratory distress at birth.^4^

Our index case had respiratory distress due to the presence of atretic posterior choanae where oral airway and oxygenation was initiated, also there was ongoing discussion with the family about tracheotomy.

Prenatal diagnostic advances have greatly improved the possibility of early detection of congenital anomalies. This gives not only an opportunity to plan and improve perinatal care but also offers the option to terminate the pregnancy for cases in which the prognosis is likely to be poor,^6^ but this was not possible in our case.

Surgical reconstruction is usually delayed until preschool years around 6 years when facial development is nearly complete but due to associated malformations management controversies regarding timing and surgical techniques.^7^

## 4 CONCLUSION

Congenital hyporhinia or partial arhinia is an extremely uncommon entity with only four cases previously reported in the literature, usually associated with other craniofacial malformations as our case which was associated with holoprosencephaly with cebocephaly which indicate a poor prognostic factor.

Nasal reconstruction during childhood is a surgical challenge, due to infrequent of this pathology and associated malformations hence management controversies regarding timing and surgical techniques.

## AUTHOR CONTRIBUTIONS

KM conceptualized and drafted the manuscript. AM reviewed the patient records. PS, DC, and PM reviewed the final script. All authors have read and approved the script.

## FUNDING INFORMATION

The author(s) received no financial support for the research, authorship, and/or publication of this article.

## CONFLICT OF INTEREST STATEMENT

The author(s) declared no potential conflicts of interest with respect to the research, authorship, and publication of this article.

## ETHICAL APPROVAL

Our institution does not require ethical approval for reporting individual cases or case series.

## CONSENT

Written informed consent was obtained from the patient to publish this report in accordance with the journal's patient consent policy.

## Data Availability

We have not shared patient's hospital record as they contain personal identification information.
